# Conditional survival in breast cancer up to 10 years in the Nordic countries

**DOI:** 10.1002/cam4.6436

**Published:** 2023-08-14

**Authors:** Frantisek Zitricky, Asta Försti, Akseli Hemminki, Kari Hemminki

**Affiliations:** ^1^ Biomedical Center, Faculty of Medicine Charles University Pilsen Pilsen Czech Republic; ^2^ Hopp Children's Cancer Center (KiTZ) Heidelberg Germany; ^3^ Division of Pediatric Neurooncology, German Cancer Research Center (DKFZ) German Cancer Consortium (DKTK) Heidelberg Germany; ^4^ Cancer Gene Therapy Group, Translational Immunology Research Program University of Helsinki Helsinki Finland; ^5^ Comprehensive Cancer Center Helsinki University Hospital Helsinki Finland; ^6^ Division of Cancer Epidemiology German Cancer Research Center (DKFZ) Heidelberg Germany

**Keywords:** conditional survival, metastasis, periodic survival, recurrence, screening, treatment

## Abstract

**Background:**

Survival in breast cancer (BC) has developed favorably but late recurrences are still a problem.

**Methods:**

We model survival data from the NORDCAN database and analyze 1‐, 5‐, and 10‐year relative survival and 5/1‐ and 10/5‐year conditional survival in BC from Denmark (DK), Finland (FI), Norway (NO), and Sweden (SE) between 1971 and 2020. Conditional survival measures survival in those who had survived year 1 to reach year 5 (5/1), or in those who had survived year 5 to reach year 10 (10/5).

**Results:**

Almost all survival metrics were best for SE but survival in all countries improved in the course of time approaching the SE levels which were 98.3% for 1‐year, 92.3% for 5‐year, and 87.8% for 10‐year survival. Conditional 10/5‐year survival, covering 5 years, was better than 5/1‐year survival, covering 4 years. A contributing factor is most likely the high rate of recurrence in period 2–5 years. The difference was observed for all countries but for DK 10/5‐year survival approached 1‐year survival and for NO and SE 10/5‐year survival was only barely better than 5/1‐year survival. The explanation to this was the excellent 10/5‐year survival in DK compared to SE and particularly to NO. Literature search suggested that the reason for the relatively low 10/5‐year survival in NO might be stagnant survival development in old patients.

**Conclusions:**

We assume that late mortality is critically limiting survival in BC and either interference with the late metastatic process or effective treatment will be key to future improvements in BC survival.

## 1 INTRODUCTION

In developed countries, some 5% of breast cancer (BC) is metastatic at the time of diagnosis (de novo metastases).^1^ However in the course of BC survival recurrent metastasis may appear with a peak incidence at about 2 years after diagnosis and the risk of metastases will remain elevated for more than 20 years.^2^ Recurrent metastases may be 2‐ to 5‐times more common than de novo metastases found at diagnosis, and they tend to carry a worse survival.^3^, ^4^, ^5^, ^6^ Treatment for BC has traditionally been surgery supported with radiotherapy which has been curative in localized disease.^7^ However as effective chemotherapies were developed toward the end of 1960s, these (including the cyclophosphamide, methotrexate, and fluorouracil combination in 1976) were eventually tested in the adjuvant setting in localized BC and the frequency of recurrences was significantly decreased, starting the era of adjuvant therapy for BC.^8^ Another adjuvant treatment for BC was endocrine therapy, based on antiestrogenic action of tamoxifen, which became a common treatment in the mid‐1980s.^7^ These were the treatments that were described in the first clinical recommendations for BC by European Society for Medical Oncology (ESMO): diagnosis, adjuvant treatment, and follow‐up of primary BC.^9^ Since then ESMO has published more extensive and targeted clinical practice guidelines for BC, primary BC guidelines introduced five BC subtypes and the recommended systemic treatment for these ranging from endocrine therapy or chemotherapy alone or in combination with anti‐HER2 therapy.^2^ Recent guidelines for metastatic BC recommended either re‐application of the therapy for primary BC or selection of novel agents depending on tumor subtypes.^6^

We assess here relative survival in female BC in the Nordic countries Denmark (DK), Finland (FI), Norway (NO), and Sweden (SE) through 50 years up to 2020 with specific aims of comparing trends in country‐specific relative survival and conditional relative survival and of poinpointing critical survival changes in years following diagnosis. The countries have organized health care largely in a similar way, they have had long‐term clinical collaboration and shared treatment guidelines (Scandinavian Breast Cancer Group, the ESMO recommendations), and national mammographic screening programs have been running in each country.^10^, ^11^ Cancer registration was started early in these countries and is generally characterized by high quality as to coverage and minimal loss to follow‐up.^12^ We obtained BC survival data from the NORDCAN database for 1‐, 5‐, and 10‐year survival, and developed conditional survival data between years 2–5, 5–10 and 2–10 years, allowing assessment of changes in survival at various intervals in the four countries. We try to correlate the observed survival changes with known developments in BC diagnostics and treatment.

## 2 METHODS

Survival data were obtained from NORDCAN database 2.0, covering years 1971 to 2020.^12^, ^13^ The database was accessed at the International Agency for Cancer (IARC) website (https://nordcan.iarc.fr/en),^14^ with a total number of female BCs, 174,175 in DK, 151,453 in FI, 114,443 in NO, and 271,653 in SE. The coverage of cancers and the follow‐up are practically complete.^12^ The available tools were used to extract data on 1‐, 5‐, and 10‐year relative survival. For 5‐year survival, NORDCAN applied the cohort survival method for the first nine 5‐year periods, and a hybrid analysis combining period and cohort survival in the last period 2016–2020; for 10‐year survival, cohort survival covered the first four 10‐year periods and the hybrid part the last 10‐year period.^15^, ^16^ Age‐standardized relative survival was estimated using the Pohar Perme estimator using national life tables in the calculation of expected rates.^17^ Age groups 0 to 89 were considered. The external age‐standardization was performed using Brenner's method, where individual observations are weighted based on age distribution of a cohort at time of diagnosis with respect to reference population.^18^ The reference age distribution was defined by the International Cancer Survival Standards (ICSSs), with weights for specific age groups in BC indicated as follows: 0–49/12, 50–59/17, 60–69/27, 70–79/29, and 80–89/15.

Conditional 5/1‐year survival was calculated by dividing the age‐standardized Pohar Perme estimate of 5‐year relative survival by the age‐standardized estimate of 1‐year relative survival; similarly, 10/5‐year conditional survival was calculated by dividing 10‐year survival estimate by 5‐year survival. All survival estimates are age‐standardized although “age‐standardization” is not always repeated.

Statistical modeling and data visualizations were performed using R statistical software (https://www.r‐project.org) in the R studio environment (https://posit.co/). We constructed Gaussian generalized additive models in Bayesian framework, with relative survival as a response variable.^19^ The predictors in GAM models included the effect of country and country‐specific non‐linear effect of time, where individual timepoints corresponded to midpoints of the respective 5‐year periods. The non‐linear effects were modeled with thin plate regression splines, where we set *k* = 5 as the parameter controlling the maximal number of knots.^20^

We run separate models for 1‐, 5‐, and 10‐year survival, respectively. The Bayesian modeling was performed using “brms” package in R.^21^ The effect of country was modeled as having Gaussian prior (mean = 0, SD = 20). The uncertainty of model input was modeled using confidence intervals provided for individual Nordcan estimates. The posterior distribution was extracted by Hamiltonian Monte Carlo sampling (2 chains of 7000 samples including 2000 warm‐ups). The models were checked in terms of convergence, effective sample size, and posterior predictive check.

For the 5/1‐year survival ratio estimation, we divided the posterior draws from the 5‐year survival model by the posterior draws from the 1‐year model to get the posterior distribution of the conditional survival and its estimated annual changes over time. The posterior distribution for 10/5‐year survival estimates was derived using analogous approach.

For all survival measures, we evaluated when the survival was changing over time with at least 95% plausibility (95% credible interval [Ci] of the first derivative of given survival measure did not cross zero for at least 5 years). “Breakpoints” identified times when the annual change of survival changed with at least 95% plausibility. This was assessed by calculation of the second derivative of the given survival measure and its 95% Ci; the “breakpoint” was defined as a peak value within at least a 3‐year interval where 95% Ci for the second derivative did not cross zero. More comprehensive approach would involve direct modeling of individual‐level hazard function with incorporating non‐linear effect of diagnostic time. However, individual‐level data were not available.

Comparisons with the US Surveillance, Epidemiology and End Results (SEER) data up to year 2018 on White women including Hispanics were done through (https://seer.cancer.gov/statistics‐network/explorer/application.html?site=1&data_type=1&graph_type=2&compareBy=sex&chk_sex_3=3&chk_sex_2=2&rate_type=2&race=1&age_range=1&hdn_stage=101&advopt_precision=1&advopt_show_ci=on&hdn_view=0&advopt_display=2#graphArea). The small difference between the SEER (any age) and our data (0–89 years) is the very old population.

## 3 RESULTS

**FIGURE 1:**
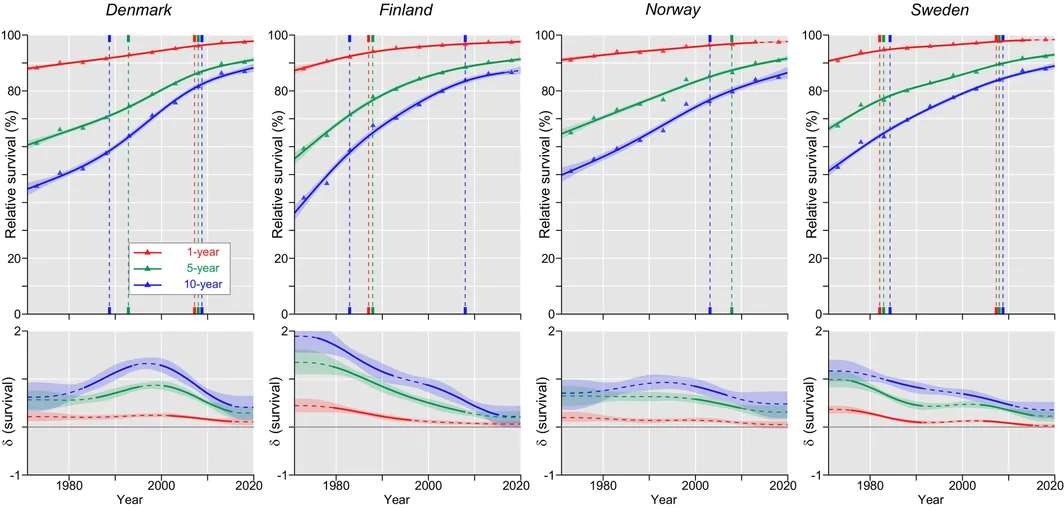
Relative 1‐, 5‐, and 10‐year survival in Denmark, Finland, Norway, and Sweden. The vertical lines mark a significant change in the survival trends (“breakpoints”), and the bottom curves show estimated annual changes in survival. The curves are solid if there is >95% plausibility of the growth or decline. Shadow areas indicate 95% credible interval. All curves are color coded (see the insert).

Relative 1‐, 5‐, and 10‐year survival in BC is shown in Figure 1, for each Nordic country. For all countries but NO there was a breakpoint for 5‐ and 10‐year survival close to 1990 after which survival increased; another but opposite breakpoint was before year 2010 for all countries. For DK, 1‐year survival was just below 90% in 1971–1975 and it approached 100% by 2016–2020. 5‐ and 10‐year survival started at a much lower level and these showed a large improvement between 1990 and 2000, when the annual change was about 1% units. All survival metrics started at a lower level in FI compared to DK but the initial survival improved rapidly (annually 2% units for 10‐year survival) and at the end matched the DK figures. All survival curves for NO and SE were almost similar, but the annual changes were largest between 1990 and 2000 in NO, and earlier in SE; the starting levels for NO and SE were well over those in DK and FI. The final difference between 5‐ and 10‐year survival was smallest in DK and largest in NO.

**FIGURE 2:**
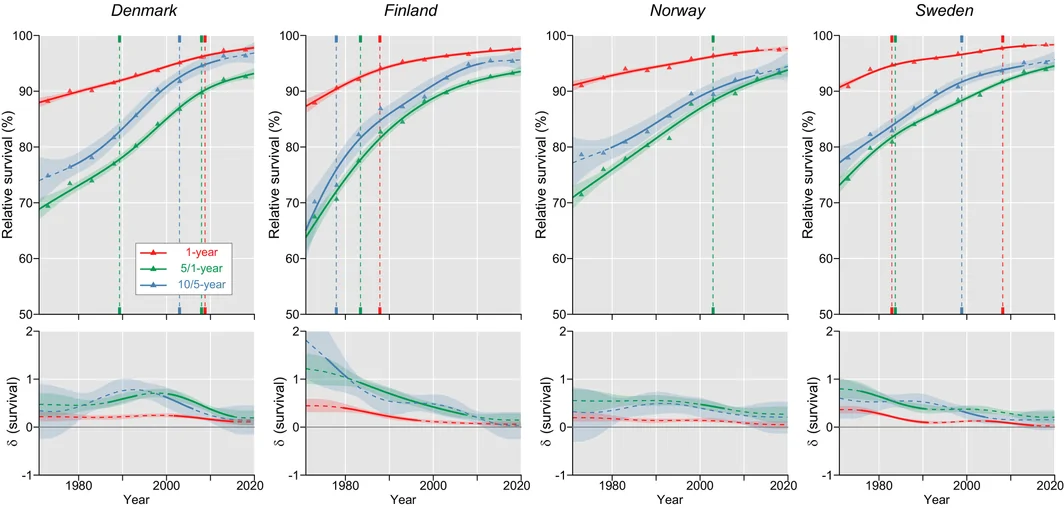
Relative 1‐year survival and conditional 5/1‐ and 10/5‐year survival in Denmark, Finland, Norway, and Sweden. The vertical lines mark a significant change in the survival trends (“breakpoints”), and the bottom curves show estimated annual changes in survival. The curves are solid if there is >95% plausibility of the growth or decline. Shadow areas indicate 95% credible interval. All curves are color coded (see the insert).

In Figure 2, we plot 1‐year survival together with conditional 5/1‐and 10/5‐year survival to allow step‐wise testing of survival in year 1, between years 2 and 5, and further between years 6 and 10. Early annual survival change in the conditional curves was almost 2% units in FI and close to 1% units in SE. The main change for conditional survival in DK and NO was between years 1990 and 2000. Note that in all countries, 10/5‐year survival was higher than 5/1‐year survival, for DK with a large and for NO with a small difference. The consequence was that in DK 1‐year and 10/5‐year survival curves approached each other while in NO they stayed far apart.

**FIGURE 3:**
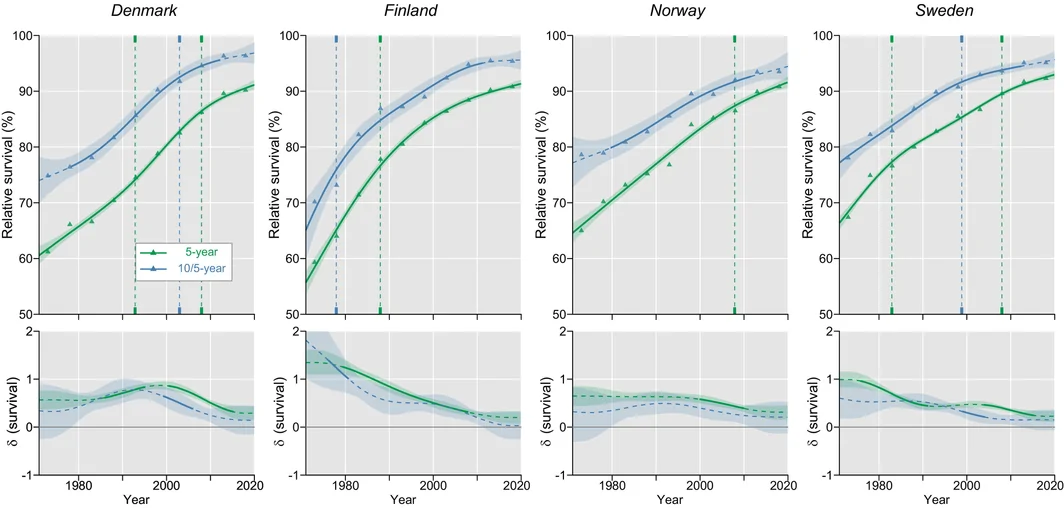
Relative 5‐year survival and conditional 10/5‐year survival in Denmark, Finland, Norway, and Sweden. The vertical lines mark a significant change in the survival trends (“breakpoints”), and the bottom curves show estimated annual changes in survival. The curves are solid if there is >95% plausibility of the growth or decline. Shadow areas indicate 95% credible interval. All curves are color coded (see the insert).

In Figure 3, we plot 5‐year survival together with conditional 10/5‐year survival. Annual survival changes for these two survival curves paralleled each other with small time‐dependent narrowing of the gap between the two. In DK, the gap remained large while in NO it was only a few % units.

Table S1 shows the exact survival data for 1‐, 5‐, and 10‐year survival in these countries; the highest final figures were 98.3%, 92.3%, and 87.8% for SE. All survival figures through all periods we highest for SE, with the exception of 1971–1975 when 1‐year survival was highest for NO. All SE survival figures were significant higher (non‐overlapping 95% CIs) than the DK ones, with the exception of the three last periods for 10‐year survival. All SE 10‐year survival figures were significantly higher than the NO ones from year 2000 onwards.

Table S2 lists the conditional survival figures for all countries. Most of these were highest for SE, but particularly for 10/5‐year survival even other countries had highest figures.

Survival data from the US SEER database up to year 2018 on White women was 97.8% (1‐year), 91.4% (5‐year), and 86.2% (10‐year).

## 4 DISCUSSION

Survival figures are usually given for 1‐ and 5‐year survival which has been historically informative to provide tangible survival estimates for most cancers and to distinguish fatal cancers from benign ones. The current survival landscape has however changed, and the “real world” cancer control in the Nordic countries has achieved the level of >60% in 5‐year survival for 84% of patients diagnosed with solid cancers.^22^ Even though 1‐ and 5‐year survival metrics are still relevant in enabling observation of short‐term temporary changes it would be useful to adopt survival metrics to 10 and 20 years into routine statistics as times to cancer cure (survival in patients reaching survival in the background population) are far longer than 5 years for most cancers.^23^, ^24^ Any single‐period survival metric has the limitation of not pinpointing the timing of survival change which however is highlighted by conditional survival.^24^ Conditional survival data differ between cancers of good and poor prognosis and only in the latter conditional survival differs extensively from non‐conditional (normal) survival.^24^ The reason is that in cancers of poor prognosis metastatic deaths are common and these usually occur shortly after diagnosis; thus, patients who survive past year 1 or longer experience better survival than all patients.^24^ In overall BC, conditional survival is only marginally better than non‐conditional survival but it increases along worse clinical stage and late recurrences.^24^, ^25^ We are here constrained by data available in NORDCAN but can describe survival between years 2 and 5 and between years 6 and 10 after diagnosis.

Relative survival development in all Nordic countries in BC has been favorable, and the large differences in 10‐year survival in 1971–1975 (11% units SE‐FI) narrowed down until 2016–2020 (1.2% units SE‐FI), in spite of more than doubling of survival (in FI). Overall, the present survival data from the Nordic countries were at the level of the US White population being about 98% for 1‐year, 91% for 5‐year and 86% for 10‐year survival. However, the timing and tempo of improvements differed between the Nordic countries. FI, starting at a low level, boosted all survival metrics in the early period; DK had the most vigorous phase from 1990 to 2000; for NO and SE, the most positive developmental phase was in the early period. DK and FI were able to narrow the difference between 5‐ and 10‐year survival toward the end more than NO and SE. As a consequence, final 10/5‐year relative survival was best in DK and worst in NO (Table S2). It is noteworthy that in all countries, and most clearly in DK, 10/5‐year survival (in 2016–2020 96.3%) was better than 5/1‐year survival (92.6%); thus, less patient died in 5 years after year 5 than in 4 years after year 1 (Figure 2). According to Figure 3, it is clear that survival through the 5 years after diagnosis was far lower than survival through the next 5 years. The most critical time for diagnosis of recurrent metastasis is after 2 years of the initial diagnosis.^2^ We do not know whether early deaths were caused by de novo or recurrent metastasis but probably both could contribute to the relatively low 5/1‐year survival. It is known that lymph‐node positive patients are at a higher risk of recurrent metastases compared to node negative patients and estrogen receptor negativity is another risk factor.^2^

Interpreting survival changes based on ecological data is difficult for any cancer but population screening for BC has introduced an additional complication because of incidence and tumor type changes and because of the large proportion of screening detected BCs.^10^, ^11^ In SE, some 70% of BCs are screening detected (https://www.cancerfonden.se/om‐cancer/undersokningar/mammografi). The first NORDCAN‐based BC study covered years 1964 to 2003.^26^ In the early period, survival in DK and FI was well below that in NO and SE. FI was able to catch up by year 2000 but DK survival remained below the other countries. In SE, BC treatment was centralized early and dedicated surgical units for BC were created; BC population screening started also relatively early in SE.^10^, ^26^ Adjuvant therapy with tamoxifen, adjuvant chemotherapy, and aromatase inhibitors were introduced early in SE and thanks to advanced diagnostic methods BC was diagnosed at an earlier stage than in NO or DK.^1^, ^27^ The fast FI improvement was ascribed to the early implementation of BC screening and advances in diagnostic and treatment. Our survival results showed that the tempo in FI improvements was highest in the 1970s, more than a decade before population screening (implemented in 1986–1989), and we believe that the improvement in FI was through emphasis of BC diagnostics, surgery, radiotherapy, and adjuvant therapy according to the above SE model.^28^, ^29^, ^30^ For DK, late implementation of BC screening and tobacco/alcohol‐related comorbidities were used as explanations for poor DK cancer survival in the early study.^26^ DK was the first Nordic country to implement a national cancer control plan in 2000, in part as response to the poor survival figures in cancers in general.^26^ The cancer plan could not influence the early positive development in DK, observed by us, which started already in the 1980s, but it may have contributed to the strong improvement in 10/5‐year survival through facilitated patient care pathways.^31^ BC treatment by surgery and radiation was highly centralized toward the end of the millennium, which probably contributed to the strong improvements in survival.^32^

For BC treatment, national guidelines were drafted, for example, in DK since 1977 which defined an anthracycline‐based regimen as a standard chemotherapy in 1995 and from 2005 included also taxanes.^32^ From 2005, the standard endocrine therapy to postmenopausal women included aromatase inhibitors; trastuzumab was included in treatment for HER2‐positive BC.^32^ These therapeutic updatings were followed in the other countries as well as condoned by the ESMO recommendations, referred to in Introduction.^33^ However the early definition of BC guidelines in DK probably helped to explain the high rate of annual improvements that we noted for the period 1990 to 2000. Why did the NO 10/5‐year survival development lose tempo compared to the other countries? We showed age‐specific survival data from an earlier version of NORDCAN when the tool was still available.^10^ In the last available period from 2002 to 2016, survival among NO women older than 79 years did not improve and it remained at over 70% while in FI it reached over 80%. In the oldest age group, also SE survival remained stagnant.^10^ The worrisome observation was that the gap between age groups did not narrow in any of the Nordic countries in the 50 year period.^10^

The NORDCAN database allows a high‐quality description of the survival experience of BC in the Nordic countries but because there is no possibility to stratify or adjust the data according to pathological or other clinically relevant variables, the generalizability of the results to other countries or even exactly between the involved countries may have limitations. However, no examples of 50‐year survival data from other countries are available, particularly if we consider general population accessibility to medical treatment. For BC, there is the additional concern of periodic difference in introduced population screening of BC which may interfere with longitudinal comparisons.

In conclusion, BC survival experience was equalized between the Nordic countries in the course of 50 years. It is encouraging news for any national health policy that in less than 30 years FI with low initial 5‐year survival in BC was able to catch up with the leading country, SE. Even though 1‐year survival is approaching 100%, 12%–15% of patients experience extra mortality up to 10 years of diagnosis. As a contributing factor, we showed that most of these deaths occurred between years 2 and 5 after diagnosis. In DK, survival between years 6 and 10 was almost as high as survival in the first year, suggesting good control of late recurrences. Future gains in BC survival depend on earliest possible detection, effective prolonged treatment and increasing focus on aged patients.^6^, ^10^ Innovative research results and methods are needed for prediction of and interference with recurrences.

## AUTHOR CONTRIBUTIONS

**Frantisek Zitricky:** Methodology (lead). **Asta Försti:** Supervision (supporting); writing – review and editing (equal). **Akseli Hemminki:** Data curation (equal); supervision (supporting); writing – review and editing (supporting). **Kari Hemminki:** Conceptualization (lead); formal analysis (lead); writing – original draft (lead).

## FUNDING INFORMATION

Supported by the European Union's Horizon 2020 research and innovation program, grant no. 856620, Jane and Aatos Erkko Foundation, Sigrid Juselius Foundation, Finnish Cancer Organizations, University of Helsinki, Helsinki University Central Hospital, Novo Nordisk Foundation, Päivikki and Sakari Sohlberg Foundation., Finnish Red Cross Blood Service, the Cooperatio Program, research area SURG and National Institute for Cancer Research—NICR (Programme EXCELES, ID Project No. LX22NPO5102), funded by the European Union—Next Generation EU.

## CONFLICT OF INTEREST STATEMENT

A.H. is shareholder in Targovax ASA. A.H. is employee and shareholder in TILT Biotherapeutics Ltd. Other authors declared no conflict of interest.

## ETHICS STATEMENT

Aggregated data from a publicly accessible database were used posing no ethical issues.

## Supporting information


Table S1.

## Data Availability

Publicly available database was used (https://nordcan.iarc.fr/en).
