# Supplementary material for: Conditional survival in breast cancer up to 10 years in the Nordic countries
Source: Cancer Med. 2023 Aug 14;12(17):17945–51. doi: 10.1002/cam4.6436 (PMC10524006; doi:10.1002/cam4.6436)
Supplement: Supplementary file 1 — Table S1. [file CAM4-12-17945-s001.pdf]

Supplementary Table 1. 1-, 5- and 10-year relative survival [95% confidence interval] in breast cancer in the Nordic countries from 1971 to 2020 according to NORDCAN. *Significant increase between the marked and the next period.

**1-year**	**Denmark**	**Finland**	**Norway**	**Sweden**
**1971-1975**	88.2[87.3-89.0]*	87.9[86.7-89.2]*	91.0[90.1-92.0]	90.8[90.3-91.4]*
**1976-1980**	90.0[89.3-90.7]	90.6[89.7-91.5]	92.4[91.6-93.2]*	93.9[93.5-94.4]
**1981-1985**	90.1[89.4-90.7]*	92.1[91.4-92.8]*	94.0[93.4-94.7]	94.7[94.3-95.1]
**1986-1990**	91.5[90.9-92.1]*	94.1[93.5-94.7]*	93.7[93.0-94.3]	95.2[94.9-95.6]
**1991-1995**	92.9[92.4-93.5]	95.3[94.8-95.9]	94.2[93.6-94.8]*	95.9[95.6-96.3]*
**1996-2000**	93.7[93.1-94.2]*	95.6[95.1-96.1]	95.8[95.3-96.3]	96.7[96.4-97.0]
**2001-2005**	95.1[94.6-95.5]*	96.3[95.8-96.7]	96.4[95.9-96.9]	97.1[96.8-97.4]*
**2006-2010**	96.1[95.7-96.5]*	96.6[96.2-97.0]*	96.6[96.1-97.1]	97.7[97.5-98.0]
**2011-2015**	97.3[97.0-97.6]	97.4[97.1-97.8]	97.5[97.1-97.9]	98.1[97.8-98.3]
**2016-2020**	97.4[97.1-97.7]	97.4[97.1-97.7]	97.4[97.0-97.8]	98.3[98.1-98.5]
**5-year**				
**1971-1975**	61.2[59.8-62.7]*	59.3[57.3-61.3]*	65.0[63.3-66.8]*	67.4[66.4-68.4]*
**1976-1980**	66.1[64.8-67.3]	64.0[62.5-65.5]*	70.2[68.6-71.7]*	74.9[74.0-75.8]
**1981-1985**	66.6[65.5-67.8]*	71.4[70.0-72.8]*	73.2[71.8-74.6]	76.6[75.7-77.5]*
**1986-1990**	70.4[69.3-71.5]*	77.8[76.5-79.1]*	75.2[73.9-76.5]	80.0[79.2-80.7]*
**1991-1995**	74.5[73.4-75.5]*	80.5[79.3-81.7]*	76.8[75.6-78.1]*	82.8[82.1-83.6]*
**1996-2000**	78.8[77.8-79.8]*	84.3[83.3-85.3]*	84.0[82.9-85.1]	85.5[84.8-86.2]
**2001-2005**	82.5[81.6-83.4]*	86.4[85.5-87.4]*	85.2[84.2-86.3]	86.7[86.1-87.4]*
**2006-2010**	86.2[85.4-87.0]*	88.4[87.6-89.2]*	86.5[85.5-87.6]*	89.6[89.0-90.2]*
**2011-2015**	89.6[88.9-90.4]	90.2[89.5-90.9]	89.9[89.0-90.8]	91.7[91.1-92.2]
**2016-2020**	90.2[89.5-90.9]	90.8[90.2-91.5]	90.8[90.0-91.7]	92.3[91.7-92.8]
**10-year**				
**1971-1975**	45.8[43.8-47.9]*	41.6[39.3-44.0]*	51.1[48.6-53.8]	52.6[51.1-54.2]*
**1976-1980**	50.5[48.7-52.4]	46.8[44.8-48.9]*	55.4[53.0-57.8]	61.6[60.1-63.1]
**1981-1985**	52.0[50.3-53.8]*	58.7[56.5-60.8]*	59.2[57.1-61.4]	63.5[62.1-64.9]*
**1986-1990**	57.5[55.8-59.3]*	67.6[65.5-69.7]	62.2[60.2-64.3]	69.5[68.3-70.8]*
**1991-1995**	63.8[62.1-65.6]*	70.2[68.2-72.2]*	65.7[63.8-67.7]*	74.4[73.2-75.7]*
**1996-2000**	71.1[69.4-72.8]*	75.0[73.1-76.9]*	75.2[73.4-77.1]	77.6[76.4-78.7]*
**2001-2005**	75.7[74.1-77.3]*	79.8[78.2-81.4]*	76.2[74.5-78.0]	80.6[79.5-81.7]*
**2006-2010**	81.5[80.0-83.0]*	83.8[82.4-85.3]	79.6[77.9-81.3]*	83.9[82.9-85.0]*
**2011-2015**	86.3[85.0-87.7]	86.1[84.8-87.4]	84.0[82.3-85.8]	87.2[86.1-88.2]
**2016-2020**	86.9[85.7-88.2]	86.6[85.5-87.8]	84.9[83.1-86.7]	87.8[86.8-88.8]

Supplementary Table 2. 5/1-, 10/5- and 10/1-year conditional relative survival in breast cancer in Nordic countries from 1971 to 2020 according to NORDCAN.

5/1-year	Denmark	Finland	Norway	Sweden
**1971-1975**	69.4	67.5	71.4	74.2
**1976-1980**	73.4	70.6	76.0	79.8
**1981-1985**	73.9	77.5	77.9	80.9
**1986-1990**	76.9	82.7	80.3	84.0
**1991-1995**	80.2	84.5	81.5	86.3
**1996-2000**	84.1	88.2	87.7	88.4
**2001-2005**	86.8	89.7	88.4	89.3
**2006-2010**	89.7	91.5	89.5	91.7
**2011-2015**	92.1	92.6	92.2	93.5
**2016-2020**	92.6	93.2	93.2	93.9
**10/5-year**				
**1971-1975**	74.8	70.2	78.6	78.0
**1976-1980**	76.4	73.1	78.9	82.2
**1981-1985**	78.1	82.2	80.9	82.9
**1986-1990**	81.7	86.9	82.7	86.9
**1991-1995**	85.6	87.2	85.5	89.9
**1996-2000**	90.2	89.0	89.5	90.8
**2001-2005**	91.8	92.4	89.4	93.0
**2006-2010**	94.5	94.8	92.0	93.6
**2011-2015**	96.3	95.5	93.4	95.1
**2016-2020**	96.3	95.4	93.5	95.1
**10/1-year**				
**1971-1975**	51.9	47.3	56.2	57.9
**1976-1980**	56.1	51.7	60.0	65.6
**1981-1985**	57.7	63.7	63.0	67.1
**1986-1990**	62.8	71.8	66.4	73.0
**1991-1995**	68.7	73.7	69.7	77.6
**1996-2000**	75.9	78.5	78.5	80.2
**2001-2005**	79.6	82.9	79.0	83.0
**2006-2010**	84.8	86.7	82.4	85.9
**2011-2015**	88.7	88.4	86.2	88.9
**2016-2020**	89.2	88.9	87.2	89.3
